# Application of nano-hydroxyapatite matrix graft in inter-vertebral fusion therapy: a meta-analysis

**DOI:** 10.1186/s12891-023-06405-x

**Published:** 2023-05-27

**Authors:** Kui Zhang, Yandong Zhu, Wenji Wang

**Affiliations:** 1 The First Clinical Medical College of Lanzhou University, Lanzhou, 730000 China; 2 Department of Orthopedics, Ninth Hospital of Xi’An, Xi’An, 710000 China; 3 Department of Orthopedic, The First Clinical Medical College of Lanzhou University, Lanzhou, 730000 China

**Keywords:** Nano-hydroxyapatite, Inter-vertebral fusion, Meta-analysis, Bone graft

## Abstract

**Objective:**

Nano-hydroxyapatite and its composites(nHA) have been widely used as grafts in inter-vertebral fusion. However, the safety and efficacy of the graft in inter-vertebral fusion is controversial. This meta-analysis aimed at evaluating the safety and efficacy of nHA and non-hydroxyapatite grafts (noHA) (autologous bone, etc.) in inter-body fusion.

**Materials and methods:**

A comprehensive search was performed in electronic database as follows: PubMed, EMBASE, the Cochrane Library, Web of Science, and China National Knowledge Internet (CNKI) from inception until October 2022. Clinical studies on the effect of nHA and noHA in spinal fusion were collected. Analysis of outcome indicators using RevMan 5.4 statistical software.

**Results:**

The meta-analysis showed that the operation time of patients who underwent inter-body fusion with nHA grafts was less than that of patients who underwent noHA (*p* < 0.05). Compared with the noHA group, the nHA group can achieve similar clinical effects in the fusion rate(OR = 1.29,95%CI: 0.88 to 1.88,*p* = 0.19),Subsidence rate(OR = 1.2,95%CI:0.44 to 3.28,*p* = 0.72), inter-vertebral space height(SMD = 0.04,95%CI:-0.08 to 0.15,*p* = 0.54),Cobb angle(SMD = 0.21,95%CI: 0.18 to 0.6,*p* = 0.21),Blood loss(SMD = -36.58,95%CI: -81.45 to 8.29,*p* = 0.11),operative time in 12 months(SMD = -5.82,95%CI: -9.98 to -1.67,*p* = 0.006) and in the final follow-up(SMD = -0.38,95%CI: -0.51 to -0.26,*p* < 0.00001),ODI(SMD = 0.68,95%CI: -0.84 to 2.19,*p* = 0.38), VAS(SMD = 0.17,95%CI: -0.13 to 0.48,*p* = 0.27) and adverse events(OR = 0.98,95%CI: 0.66 to 1.45,*p* = 0.92), and the differences are not statistically significant.

**Conclusion:**

This meta-analysis suggests that nHA matrix grafts are similar to noHA grafts in the safety and efficacy of spinal reconstruction, and are an ideal material for inter-vertebral bone grafting.

**Supplementary Information:**

The online version contains supplementary material available at 10.1186/s12891-023-06405-x.

## Introduction

Inter-body fusion is a routine operation for the treatment of spinal degenerative diseases [1]. It achieves clinical effects such as correcting deformity, reconstructing spinal stability, and relieving pain by accelerating bone fusion [2]. Although inter-vertebral fusion is widely used in clinical practice and mature in technology, different grafts used in the operation have different effects on the functional improvement, cone sedimentation rate and cone fusion rate of patients undergoing fusion surgery [3–9].

Nano-hydroxyapatite (nHA) is the main mineral in natural bones. Because of its similar chemical and physical properties to human bones, good biological activity and bone conductivity, it has a broad environment in medical applications [10, 11]. nHA matrix graft is a new type of bone reconstruction and bone repair material in recent years. It has been widely used in clinical practice and has achieved good clinical results [12]. Relevant literature studies have shown that nHA grafts have stable biomechanics, similar elastic modulus to the bone tissue, and good biocompatibility [13]. In patients with inter-vertebral fusion, it has the characteristics of less complication, high fusion rate and good bone resorption. It is a widely used bone graft filling material [14, 15]. At present, there have been some clinical studies on the safety and efficacy of nHA grafts and noHA matrix grafts in inter-vertebral fusion [14, 16–26]. Some research conclusions [14, 19, 23, 26, 27] is controversial, but no relevant systematic analysis has been found to demonstrate these conclusions. Therefore, this study collected relevant clinical studies, to take a meta-analysis method to analyze the safety and efficacy of nHA matrix graft in inter-body fusion.

## Material and methods

### Data sources and searches

This study was performed using a prior established protocol, and was conducted in accordance with the Preferred Reporting Items for Systematic Reviews and Meta-Analyses (PRISMA) Extension Statement for systematic reviews incorporating network meta-analyses.

An extensive search of PubMed, Web of Science, China National Knowledge Internet (CNKI), EMBASE, and the Cochrane Library from the establishment date of the database to October 2022 was used the following key search terms: “hydroxyapatite”, “bone graft”, “Bone Transplantation”, “spine”, “lumbar vertebrae”, “thoracic vertebra”, “cervical vertebra”, “Arthrodesis”. Lists of references cited in relevant systematic reviews and included trials were also screened. Two investigators conducted the search independently.

### Inclusion and exclusion criteria

Inclusion criteria were as follows:(i) Retrospective case–control studies (RPCT), randomised controlled trials (RCT), prospective case–control studies (PCCT); (ii) Patients must undergo inter-vertebral fusion surgery; (iii) Patients were followed up for more than 24 weeks; (iv) sufficient published data to estimate odds ratio (OR), or standardized mean difference (SMD) with a 95% confidence interval (CI).

Studies were considered exclusion for this study if they met the following criteria:(i) meeting proceedings, abstracts, letters, editorials, reviews or case reports; (ii) Research without a full-text (iii) Studies lacking comparable results; (iv) no outcomes of interests; and (v) repeated reports.

### Study selection

Two researchers independently developed the search strategy and sifted through all the initial literature results. Initial literature screening was performed by evaluating the titles and abstracts of the studies. The final two researchers determined the final inclusion literature by reading the full text according to establish inclusion and exclusion criteria. Disagreements on inclusion were resolved through discussion and consensus.

### Outcome measures

Two researchers independently extracted available data from the included literature for analysis by reading the full text. The basic characteristics of the study (author, publication date, design type, publication country), demographic data of patients (age, sample size, follow-up time, surgical method) and the main outcome indicators of the study (fusion rate, subsidence rate, inter-vertebral space height (IH), Cobb angle, blood loss, operative time, the Oswestry Disability.

Index (ODI), the Visual Analogue Scale score (VAS), adverse events) were extracted from the final included study.

### Data quality assessment

The two researchers independently assessed the quality of the included studies based on the Newcastle–Ottawa Scale (NOS), which covered three areas: object selection, condition suitability and exposure. The highest score of each study was nine, and ≥ 6 were considered to be superior-quality studies. Disagreements regarding inclusion were resolved through discussion and consensus.

### Data synthesis and analysis

Statistical analyses were performed using Review Manager (RevMan) [Computer program]. Version 5.4. The Cochrane Collaboration, 2020. The results for the dichotomous effect size are computed using the OR and the continuous effect size results from SMD.A 95% CI were determined for each effect size. Chi-squared tests and I-squared (I^2^) statistics were tested for the heterogeneity in each study. The heterogeneity of each study was tested by Chi-squared tests and I-squared (I^2^) statistics. When *p* > 0.1 and I^2^ < 50%, with low heterogeneity, the analysis was performed using a fixed effect model. Instead, a random effect model was applied to the analysis. Sensitivity analysis was performed by excluding some studies and calculating the effect results.

## Results

**Fig. 1:**
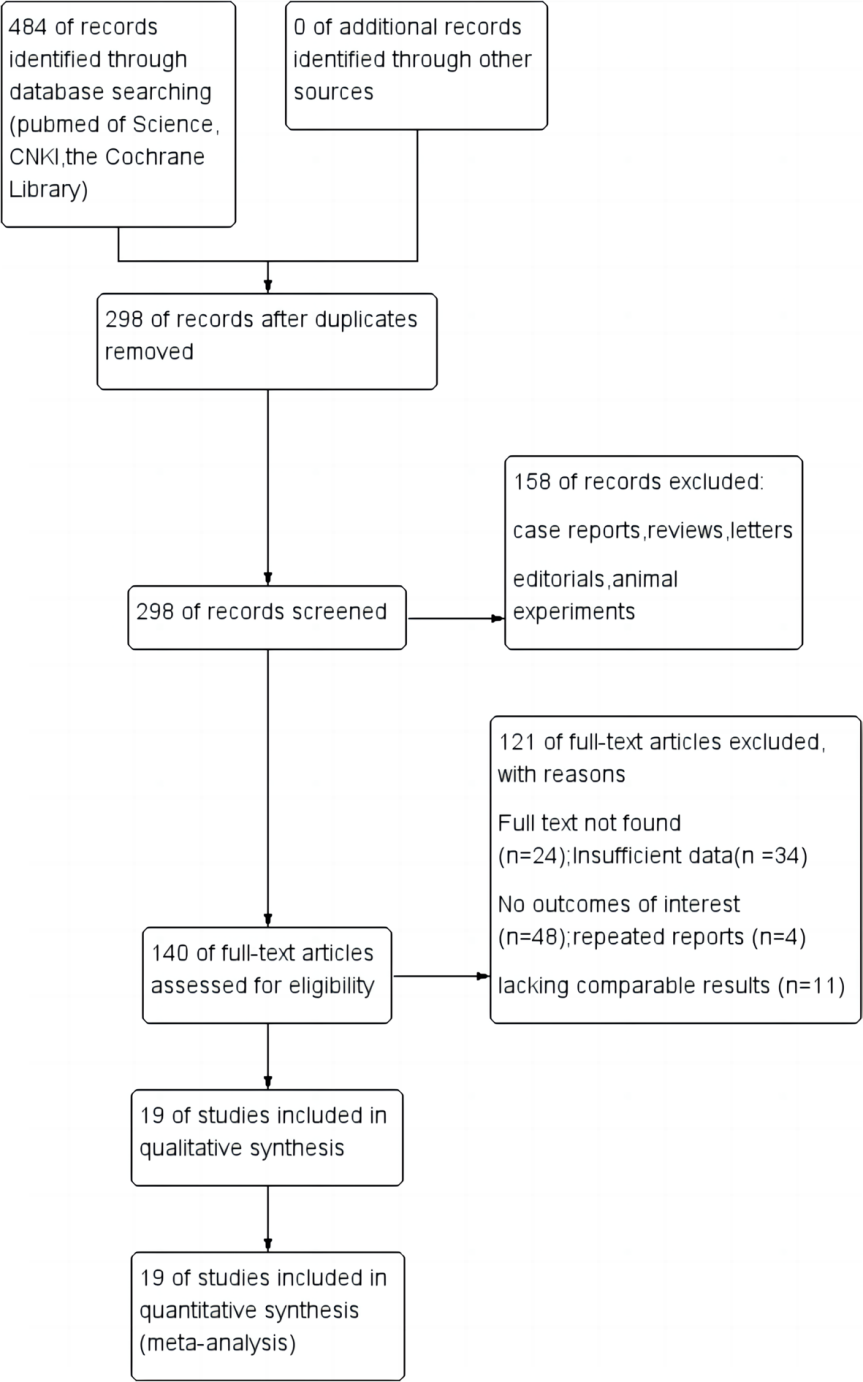
PRISMA flow diagram of study selection for the current meta-analysis

A total of 484 relevant studies were retrieved from the relevant databases. After removing duplicates, 298 articles remained. Then, 158 studies were discarded by title and summary reading. Through full-text reading of the remaining 140 papers, 121 studies were excluded due to incomplete full-text, inconsistencies, or missing results on the effect of the study. A total of 19 studies [4, 14, 16, 18–33] were included in the meta-analysis (Fig. 1). In addition, all included studies can be considered to be of relatively high quality based on the results of the NOS rating scale. See Table 1 for more detailed basic features.

**Table 1:** Basic characteristics of enrolled studies

Author/year	Country	Study design	Age (years)	Sample Size nHA/noHA(n)	Type of surgery	Outcome indicators	Follow-up time (months)	NOS score
D.Neen 2006 [16]	UK	PCCT	49/48	50/50	PLIF	①,⑨	24	7
E.Dawson 2009 [19]	USA	PCCT	55.9/56.9	25/21	PLDIF	①,⑤,⑥,⑦,⑨	24	8
X.H.Liu 2012 [30]	China	PCCT	51.4/52.4	31/26	ACDF	①,②,③,④,⑥,⑨	24	7
J.X.Liang 2018 [29]	China	PCCT	50.5/52.5	124/50	ACDF	①,②,③,④,⑤,⑥,⑦,⑧	52	8
C.Zhu 2021 [26]	China	PCCT	54.5/55.3	32/32	TLIF	①,③,④,⑤,⑥,⑧	47	7
T.Yoshii 2021 [25]	Japan	PCCT	70.2/73.8	46/46	PLIF	①,⑨	24	8
J.Delecrin 2000 [32]	France	RCT	18.2/17.5	28/30	PLIF	④,⑤,⑥,⑨	24	8
J.R.McConnell 2003 [33]	UK	RCT	47/47	13/16	ACDF	①,②,⑨	24	8
P.Korovessis 2005 [27]	Greece	RCT	58/61	20/19	PLDIF	⑤,⑥,⑦,⑧	48	8
J.R.Dimar 2009 [14]	USA	RCT	53.2/52.3	239/224	PLDIF	①,⑤,⑥,⑨	24	7
N.H.vonderHoeh 2017 [22]	Germany	RCT	64.3/65.6	24/24	TLIF	①,⑦,⑧,⑨	12	8
J.H.Cho 2017 [21]	Korea	RCT	64.9/62	42/51	PLDIF	①,⑦,⑧,⑨	6	8
M.Rickert 2019 [23]	Germany	RCT	60.6/66.1	20/20	ALIF	①,⑧,⑨	12	8
W.Chen 2020 [28]	China	RCT	48.6/48	19/10	TLIF	①,③,④,⑤,⑥,⑦,⑨	36	7
B.L.Ma 2016 [31]	China	RCT	49.2/48	20/10	TLIF	①,④,⑤,⑥,⑦,⑨	12	7
W.C.Chang 2009 [18]	China	RPCT	58.5/51.39	22/23	ACDF	①,⑨	6	7
Q.x.Deng 2016 [20]	China	RPCT	53.2/53.6	124/142	TLIF	①,②,③,④,⑤,⑥,⑧,⑨	47	8
B. Hu 2019 [4]	China	RPCT	52.5/51.3	47/51	ACDF	①,②,③,④,⑤,⑥,⑧,⑨	84	7
Tayfun Cakir 2021 [24]	Turkey	RPCT	61.4/66.1	54/51	PLIF	③,⑦,⑧,⑨	60	8

*PCCT* Prospective case–control study, *RCT* Randomized controlled study, *RPCT* Retrospective case–control study, *PLIF* Posterior lumbar intertransverse fusion, *PLDIF* Posterior lumbar decompression and intertransverse fusion, *ACDF* Anterior cervical decompression and fusion, *TLIF* Transforaminal lumbar inter-body fusion. ①fusion rate,②Subsidence rate,③inter-vertebral space height,④Cobb angle,⑤blood loss,⑥operative time,⑦ODI⑧VAS,⑨adverse events

### Fusion rate

The follow-up time for the fusion rate varied widely, ranging from 6 to 84 months. Therefore, the subgroup analysis is based on the time period.​

**Fig. 2:**
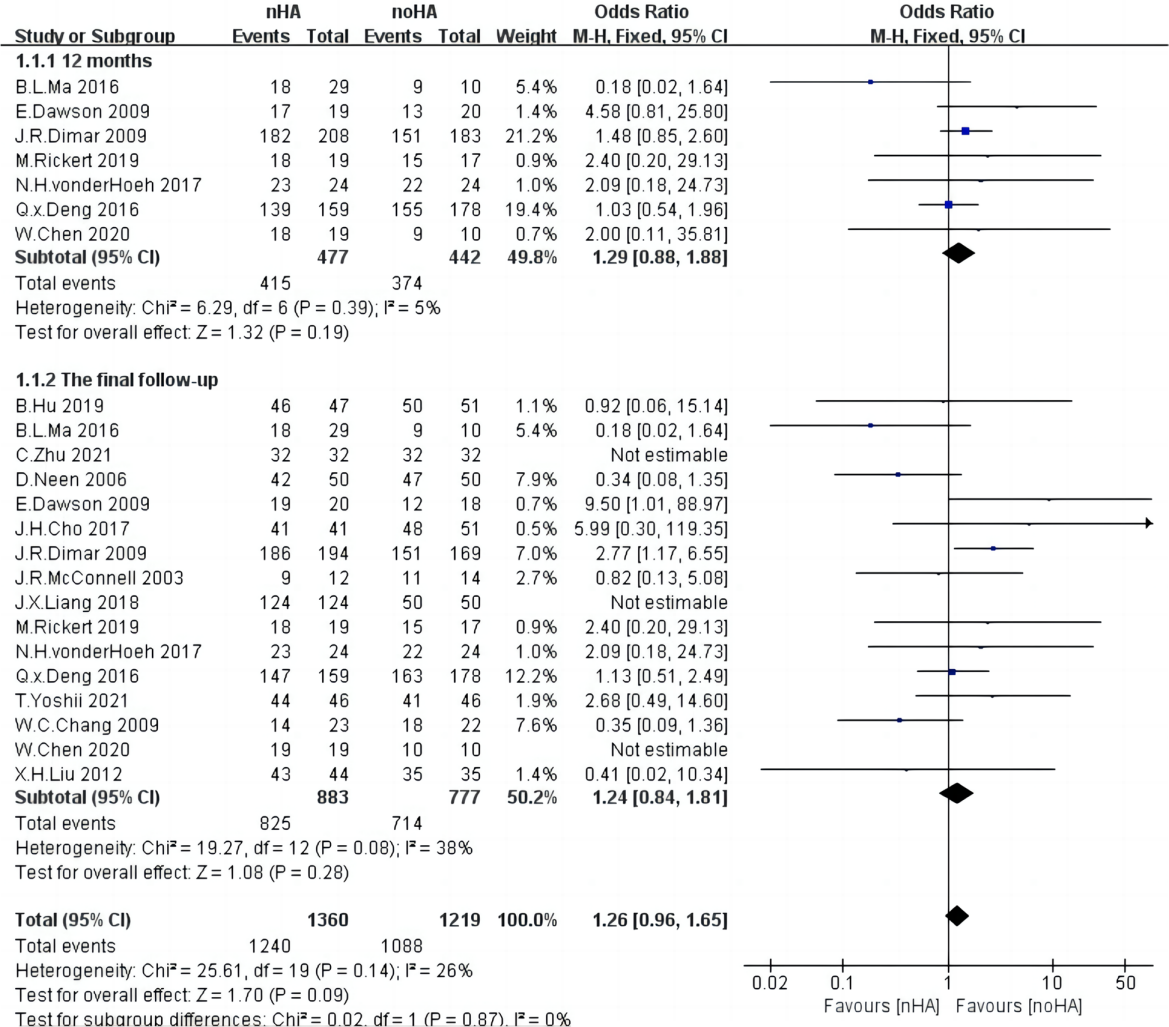
The forest plot of fusion rate of nHA group versus noHA group

Fusion rates at 12 months after surgery from seven studies [14, 19, 20, 22, 23, 28, 31] including 919 patients were available for analysis. A fixed-effect model was adopted as the heterogeneity among included studies was relatively low (Chi^2^ = 6.29,* p* = 0.39, I^2^ = 5%) (Fig. 2). As a result, the amount of fusion rate in nHA patients was no significant difference in noHA patients (OR = 1.29,95%CI: 0.88 to 1.88,* p* = 0.19).

Data on fusion rates at final follow-up after surgery were assessed in 16 studies [4, 14, 16, 18–23, 25, 26, 28–31, 33], including 1660 patients. Low heterogeneity was observed across each study (Chi^2^ = 19.27,* p* = 0.08, I^2^ = 38%), so the fixed-effect model was adopted. Again, the results exhibited no significant difference in the fusion rate between the two groups at the final follow-up (OR = 1.24,95%CI: 0.84 to 1.81,* p* = 0.28) (Fig. 2).

### Subsidence rate

**Fig. 3:**
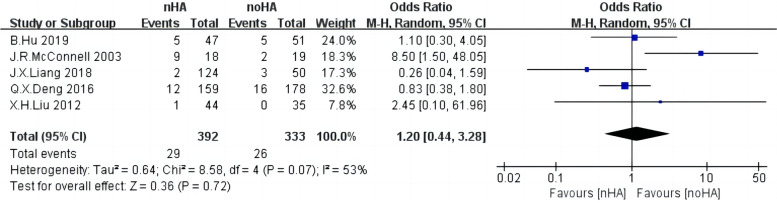
The forest plot of Subsidence rate of nHA group versus noHA group

A total of five included studies [4, 20, 29, 30, 33] with 725 patients examined the settling rates in both groups. The random-effect model was then employed because of high heterogeneity (Chi^2^ = 8.58,* p* = 0.0.07, I^2^ = 53%) (Fig. 3). It was not significantly different between the two groups (OR = 1.2,95%CI:0.44 to 3.28,* p* = 0.72).

### Inter-Vertebral space height (IH)

**Fig. 4:**
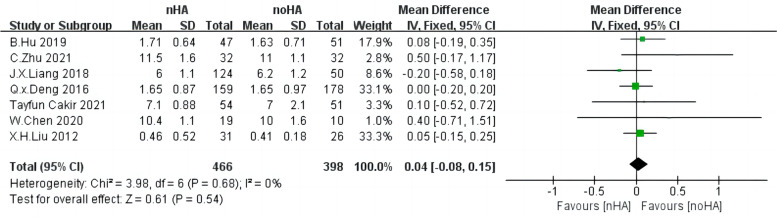
The forest plot of inter-vertebral space height of nHA group versus noHA group

Seven studies [4, 20, 24, 26, 28–30] consisting of 864 patients documented IH. The heterogeneity among included studies was relatively low (Chi^2^ = 3.98,* p* = 0.68, I^2^ = 0%) and the fixed-effect model was used for analysis (Fig. 4). It was not significantly different between the two groups (SMD = 0.04,95%CI: -0.08 to 0.15,* p* = 0.54).

### Cobb angle

**Fig. 5:**
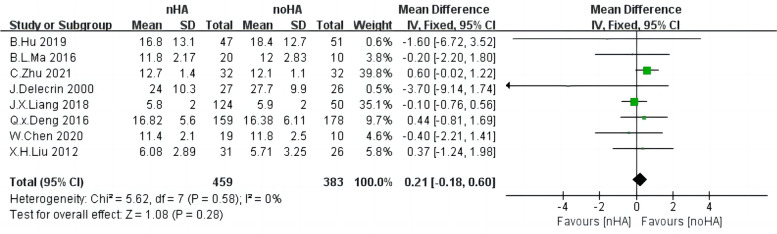
The forest plot of Cobb angle of nHA group versus noHA group

Regarding the Cobb angle, 842 patients from eight studies [4, 20, 26, 28–32] were pooled in the analysis. There was low heterogeneity across each study (Chi^2^ = 5.62,* p* = 0.58, I^2^ = 0%) and we used the fixed-effect model (Fig. 5). No significant difference was found between the nHA and the noHA groups (SMD = 0.21,95%CI: 0.18 to 0.6,* p* = 0.21).

### Blood loss

**Fig. 6:**
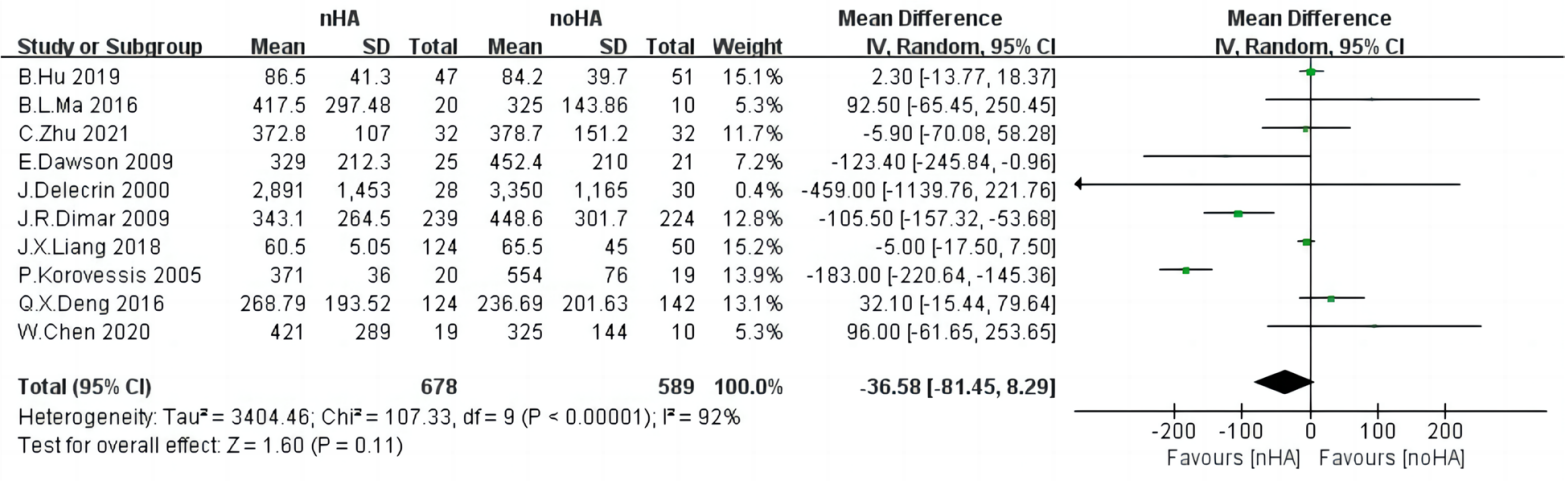
The forest plot of Blood loss of nHA group versus noHA group

With respect to blood loss during surgery, ten studies [4, 14, 19, 20, 26–29, 31, 32] consisting of 1267 patients were pooled for this outcome using a random effect model due to high heterogeneity. (Chi^2^ = 107.33,* p* < 0.0001, I^2^ = 92%) (Fig. 6). Again, the results did not show a significant difference in blood loss between the two groups (SMD = -36.58,95%CI: -81.45 to 8.29,* p* = 0.11).

### Operative time

Regarding the operation time, since the data included in the analysis were taken in minutes and hours, the data were subgroup and analyzed in subgroups by Minutes and Hours, respectively.

**Fig. 7:**
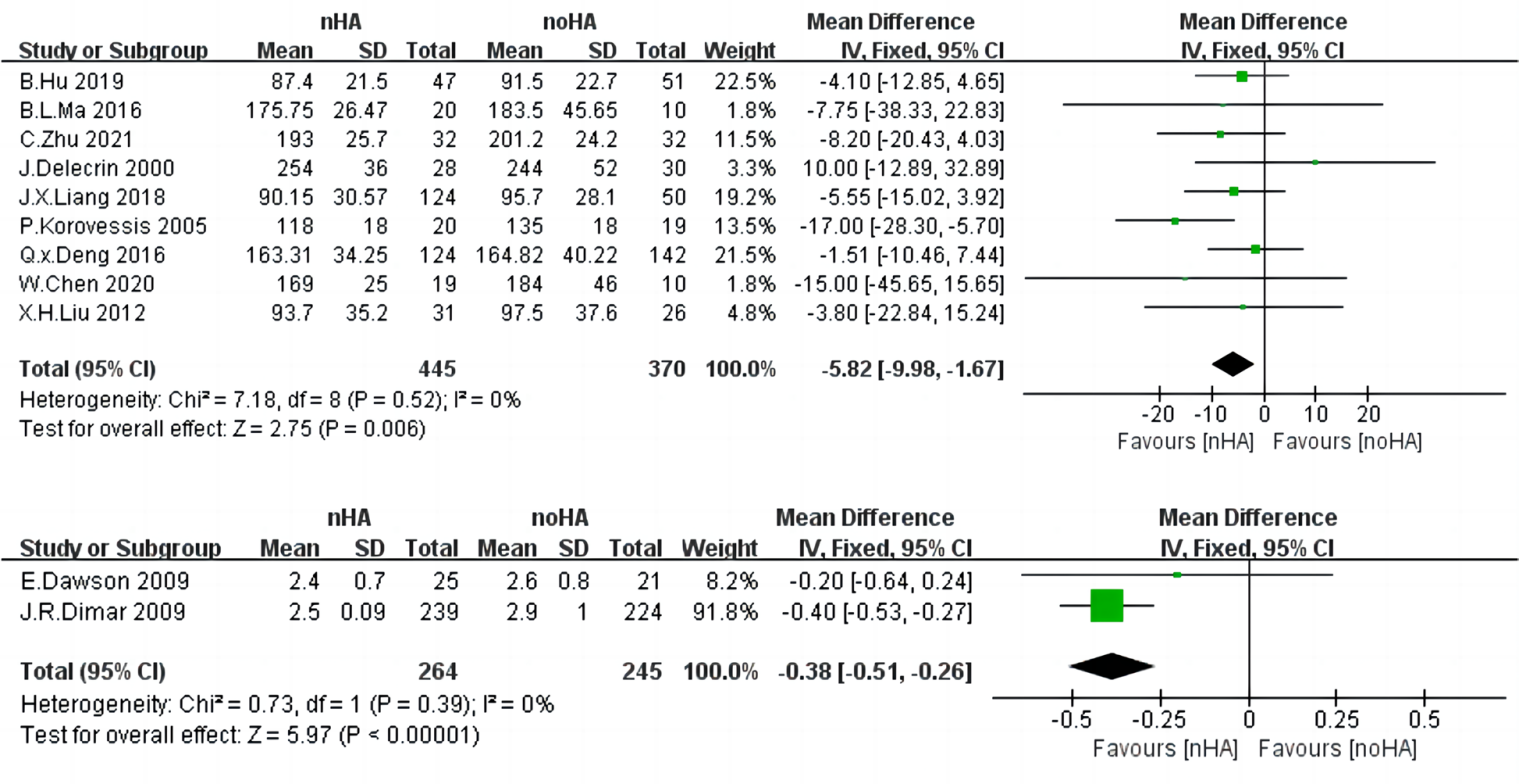
The forest plot of operative time of nHA group versus noHA group

A total of nine inclusion studies [4, 20, 26–32] involving 815 patients in the Minutes group and two inclusion studies [14, 19] involving 509 patients in the Hours group examined the timing of surgery. There is low heterogeneity among the studies (Chi^2^ = 7.18,* p* = 0.52, I^2^ = 0%) and (Chi^2^ = 0.73,* p* = 0.39, I^2^ = 0%), the fixed-effect model was used for analysis (Fig. 7). The operation time of nHA patients was significantly less than the time observed in noHA patients (SMD = -5.82,95%CI: -9.98 to -1.67,* p* = 0.006) and (SMD = -0.38,95%CI: -0.51 to -0.26,* p* < 0.00001).

### ODI

**Fig. 8:**
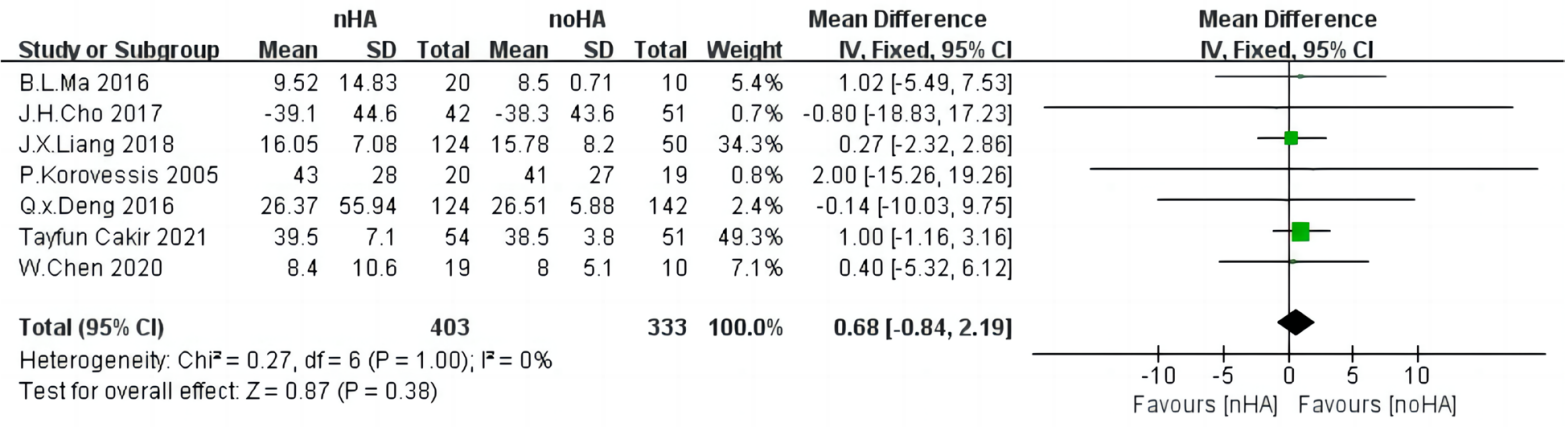
The forest plot of ODI nHA group versus noHA group. ODI: the Oswestry Disability Index

Regarding ODI, 736 patients from seven studies [20, 21, 24, 27–29, 31] were pooled in the analysis. There was low heterogeneity across each study (Chi^2^ = 0.27,* p* = 1, I^2^ = 0%) and we used the fixed-effect model (Fig. 8). The results exhibited no significant difference in the ODI between the two groups (SMD = 0.68,95%CI: -0.84 to 2.19,* p* = 0.38).

### VAS

**Fig. 9:**
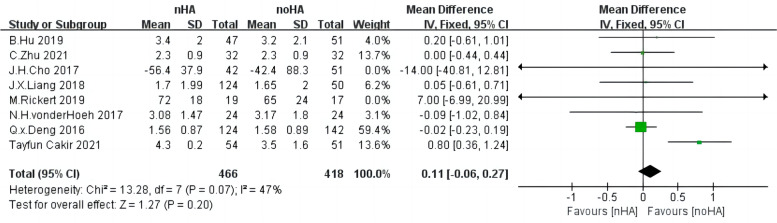
The forest plot of VAS of nHA group versus noHA group. VAS: the Visual Analogue Scale score

Eight studies [4, 20–24, 26, 29] consisting of 884 patients documented VAS. The fixed-effect model was employed because of low heterogeneity (Chi^2^ = 13.28,* p* = 0.07, I^2^ = 47%). No significant difference was found between nHA and noHA groups (SMD = 0.17,95%CI: -0.13 to 0.48,* p* = 0.27) (Fig. 9).

### Adverse events

**Fig. 10:**
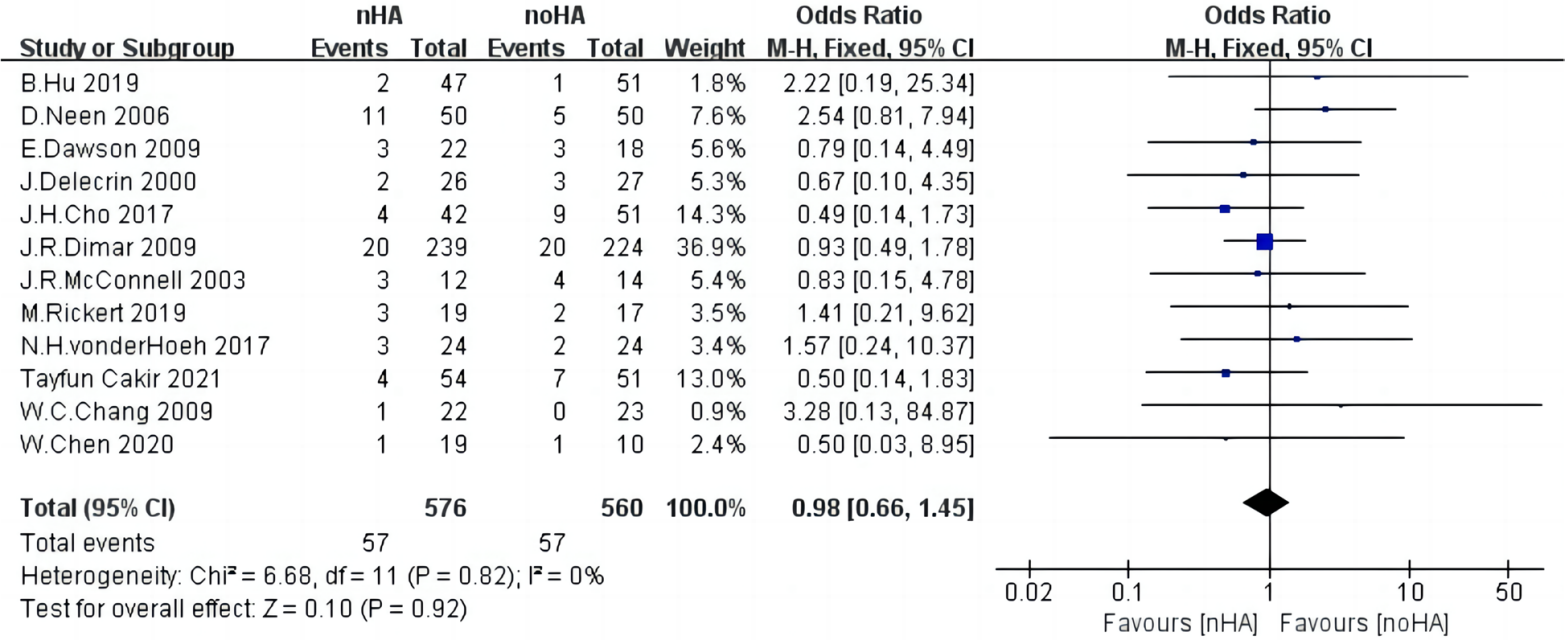
The forest plot of adverse events of nHA group versus noHA group

For adverse events, 1,136 patients from 12 studies [4, 14, 16, 18, 19, 21–24, 28, 32, 33] were pooled in the analysis. A fixed-effect model was adopted as the heterogeneity among included studies was relatively low (Chi^2^ = 6.68,* p* = 0.82, I^2^ = 0%) (Fig. 10). As a result, the amount of fusion rate in nHA patients was significantly less than that in noHA patients (OR = 0.98,95%CI: 0.66 to 1.45,* p* = 0.92).

## Discussion

nHA is the main mineral in natural bone. Due to its excellent mechanical properties, biocompatibility, and similar chemical and physical properties to human bone, it has attracted considerable attention in the preparation of prosthetic implants, scaffolds, and artificial bone cement [34]. However, the individual application of nHA is limited by its poor mechanical properties, and thus its application is severely limited. With the development of bio-engineering techniques and materials science, nHA can be combined with a variety of alternative materials to obtain composites with high strength and elevated osteogenic activity, which is a critical direction for bone tissue engineering research. At present, nHA is mainly combined with the following materials [35–37]: bioactive factors, synthetic polymer materials (polyhydroxy glycolic acid, polyetheretherketone, polyethylene, polylactic acid, polyamide, etc.), natural polymer materials (cellulose, silk fibroin, dextran, collagen, chitosan, etc.), and it has also been reported in literature that nHA can be combined with antibiotics, antitumor drugs, bone marrow mesenchymal stem cells and insulin-like growth factors to obtain the desired specific function. The nHA compound has excellent mechanical and biological properties. It has clear advantages over pure nHA materials and has great potential for applications in bone tissue engineering.

In this study, we compared intra- and post-operative clinical and imaging outcomes of nHA matrix composite bone grafts and noHA grafts in inter-body fusion. Spinal fusion was performed in studies involving either the cervical or thoracic or lumbar vertebra. Patients' ages varied widely among the included studies, but there was no significant difference between the experimental and control groups within each study. Operation time and intraoperative blood loss are critical indicators that reflect the safety of the surgery. Some studies [20, 28, 31] have shown that intraoperative blood loss is greater in nHA than in the control group, which may be due to the surgeon's master's degree in surgical skills. Excessive intraoperative bleeding may lead to haemorrhagic shock and damage to vital organs, which can be life-threatening in severe cases. Multiple experiments included in the meta-analysis showed that inter-body fusion did not significantly increase the risk of major intraoperative bleeding in the nHA group. This conclusion is controversial due to the large heterogeneity among the included studies.

In general, the longer the procedure, the higher the risk of intraoperative complications such as asphyxia and anesthesia accidents. Most of the included studies [4, 20, 26, 28–32] showed that there was no statistical difference in the operating time between the two groups, but meta-analysis found that the operation time of the experimental group was significantly less than that of the control group, indicating that the operation time of nHA matrix graft was less and it was safer for patients undergoing surgery. This study used the incidence of adverse events as a measure of postoperative safety and found that interbody fusion with nHA matrix grafts did not significantly increase the incidence of postoperative complications, consistent with Cakir's and Chen's findings [24, 28].

It has been shown that the different graft materials used in spinal fusion surgery can directly affect bone graft fusion rate, inter-vertebral space height and fusion segment curvature recovery [38, 39]. The results of this meta-analysis showed that: Two different materials of graft showed similar fusion rate and collapse rate, this could be due to these two kinds of material of graft has similar elastic modulus, and both by increasing the friction between the graft and endplate and dispersed pressure on the surface of the implant to prevent graft migration and sinking, help maintains the height of the inter-vertebral fusion segments and curvature fusion segments, to achieve the stability of the cone segments, it can be inferred that the nHA matrix graft has good biomechanical properties. There was no significant difference in VAS scores and ODI between the nHA and noHA groups. Overall, the meta-analysis of each test metric concluded that nHA and its related materials have stable therapeutic effects and clear advantages in terms of inter-body fusion, short operating time, high conical fusion rate and low incidence of adverse events, suggesting that nHA matrix composites are a safe and effective biomaterial.

In this meta-analysis, there is heterogeneity between nHA and noHA groups in the research of Subsidence rate. ​The study of J.R. McConnell [33] was found to be a source of heterogeneity, which was reduced after exclusion without a change in conclusions. There was inter-study heterogeneity in the analysis of surgical blood loss between the two groups and each study was excluded on a case-by-case basis. Unfortunately, we do not find which study is responsible for the elevated heterogeneity. It may be the surgical skills and clinical experience of different surgeons that lead to the correlation rather than the final extracted data, thus we may not be able to find its source. In the above meta-analysis, we used a random effects model, and the results are considered reliable.

## Conclusions

This study investigated the safety and efficacy of nHA matrix grafts and noHA grafts in spinal reconstruction. The results showed that the two regimens had similar clinical efficacy and safety. In addition, patients who underwent fusion with the nHA material had shorter surgery times and did not experience an increase in the amount of surgical bleeding or the incidence of risk events compared to the noHA graft group. There was no significant difference between the two groups in clinical outcomes in terms of VAS and ODI scores. The nHA matrix graft is an ideal alternative to inter-vertebral support bone grafts. However, the results may be biased due to the different clinical design types, aetiology and spinal surgery segments included in the study. Additional large-sample, multi-center, high-quality clinical trials should be encouraged to further validate the safety and efficacy of nHA matrix graft in spinal reconstruction.

## Supplementary Information

**Additional file 1: Supplementary table.** The Meta-Analysis Literature Search Strategy (PubMed).

## Data Availability

Our raw data are presented in the supplementary file. The datasets used and/or analysed during the current study available from the corresponding author (Wenji Wang) on reasonable request.
