# Supplementary material for: Application of nano-hydroxyapatite matrix graft in inter-vertebral fusion therapy: a meta-analysis
Source: BMC Musculoskelet Disord. 2023 May 27;24:427. doi: 10.1186/s12891-023-06405-x (PMC10224592; doi:10.1186/s12891-023-06405-x)
Supplement: Supplementary file 1 — Additional file 1: Supplementary table. The Meta-Analysis Literature Search Strategy (PubMed). [file 12891_2023_6405_MOESM1_ESM.docx]

**Supplementary table.The Meta-Analysis Literature Search Strategy (PubMed)**

| Search number | Query | Sort By | Filters | Search Details | Results | Time |
| --- | --- | --- | --- | --- | --- | --- |
| 21 | (((("Arthrodesis"[Mesh]) OR (Arthrodesis)) AND ((((((("Spine"[Mesh]) OR (spine fracture)) OR (spine)) OR (lumbar vertebrae)) OR (thoracic vertebra)) OR ("Thoracic Vertebrae"[Mesh])) OR ("lumbar Vertebrae"[Mesh]))) AND ((((bone graft) OR ("Bone Transplantation"[Mesh])) OR (bone grafting)) OR (Bone Transplantation))) AND (("Hydroxyapatites"[Mesh]) OR (Hydroxyapatites)) | Most Recent |  | ("Arthrodesis"[MeSH Terms] OR ("arthrodesed"[All Fields] OR "arthrodesing"[All Fields] OR "Arthrodesis"[MeSH Terms] OR "Arthrodesis"[All Fields] OR "arthrodese"[All Fields] OR "arthrodeses"[All Fields])) AND ("Spine"[MeSH Terms] OR ("spinal fractures"[MeSH Terms] OR ("spinal"[All Fields] AND "fractures"[All Fields]) OR "spinal fractures"[All Fields] OR ("Spine"[All Fields] AND "fracture"[All Fields]) OR "spine fracture"[All Fields]) OR ("Spine"[MeSH Terms] OR "Spine"[All Fields] OR "spines"[All Fields] OR "spine s"[All Fields]) OR ("lumbar Vertebrae"[MeSH Terms] OR ("lumbar"[All Fields] AND "vertebrae"[All Fields]) OR "lumbar Vertebrae"[All Fields]) OR ("Thoracic Vertebrae"[MeSH Terms] OR ("thoracic"[All Fields] AND "vertebrae"[All Fields]) OR "Thoracic Vertebrae"[All Fields] OR ("thoracic"[All Fields] AND "vertebra"[All Fields]) OR "thoracic vertebra"[All Fields]) OR "Thoracic Vertebrae"[MeSH Terms] OR "lumbar Vertebrae"[MeSH Terms]) AND ("Bone Transplantation"[MeSH Terms] OR ("bone"[All Fields] AND "transplantation"[All Fields]) OR "Bone Transplantation"[All Fields] OR ("bone"[All Fields] AND "graft"[All Fields]) OR "bone graft"[All Fields] OR "Bone Transplantation"[MeSH Terms] OR ("Bone Transplantation"[MeSH Terms] OR ("bone"[All Fields] AND "transplantation"[All Fields]) OR "Bone Transplantation"[All Fields] OR ("bone"[All Fields] AND "grafting"[All Fields]) OR "bone grafting"[All Fields]) OR ("Bone Transplantation"[MeSH Terms] OR ("bone"[All Fields] AND "transplantation"[All Fields]) OR "Bone Transplantation"[All Fields])) AND ("Hydroxyapatites"[MeSH Terms] OR ("durapatite"[MeSH Terms] OR "durapatite"[All Fields] OR "hydroxyapatite"[All Fields] OR "Hydroxyapatites"[MeSH Terms] OR "Hydroxyapatites"[All Fields])) | 194 | 23:24:19 |
| 20 | ("Arthrodesis"[Mesh]) OR (Arthrodesis) | Most Recent |  | "Arthrodesis"[MeSH Terms] OR "arthrodesed"[All Fields] OR "arthrodesing"[All Fields] OR "Arthrodesis"[MeSH Terms] OR "Arthrodesis"[All Fields] OR "arthrodese"[All Fields] OR "arthrodeses"[All Fields] | 45,023 | 23:22:44 |
| 19 | (((((("Spine"[Mesh]) OR (spine fracture)) OR (spine)) OR (lumbar vertebrae)) OR (thoracic vertebra)) OR ("Thoracic Vertebrae"[Mesh])) OR ("lumbar Vertebrae"[Mesh]) | Most Recent |  | "Spine"[MeSH Terms] OR ("spinal fractures"[MeSH Terms] OR ("spinal"[All Fields] AND "fractures"[All Fields]) OR "spinal fractures"[All Fields] OR ("Spine"[All Fields] AND "fracture"[All Fields]) OR "spine fracture"[All Fields]) OR ("Spine"[MeSH Terms] OR "Spine"[All Fields] OR "spines"[All Fields] OR "spine s"[All Fields]) OR ("lumbar Vertebrae"[MeSH Terms] OR ("lumbar"[All Fields] AND "vertebrae"[All Fields]) OR "lumbar Vertebrae"[All Fields]) OR ("Thoracic Vertebrae"[MeSH Terms] OR ("thoracic"[All Fields] AND "vertebrae"[All Fields]) OR "Thoracic Vertebrae"[All Fields] OR ("thoracic"[All Fields] AND "vertebra"[All Fields]) OR "thoracic vertebra"[All Fields]) OR "Thoracic Vertebrae"[MeSH Terms] OR "lumbar Vertebrae"[MeSH Terms] | 291,619 | 23:22:28 |
| 18 | (((bone graft) OR ("Bone Transplantation"[Mesh])) OR (bone grafting)) OR (Bone Transplantation) | Most Recent |  | "Bone Transplantation"[MeSH Terms] OR ("bone"[All Fields] AND "transplantation"[All Fields]) OR "Bone Transplantation"[All Fields] OR ("bone"[All Fields] AND "graft"[All Fields]) OR "bone graft"[All Fields] OR "Bone Transplantation"[MeSH Terms] OR ("Bone Transplantation"[MeSH Terms] OR ("bone"[All Fields] AND "transplantation"[All Fields]) OR "Bone Transplantation"[All Fields] OR ("bone"[All Fields] AND "grafting"[All Fields]) OR "bone grafting"[All Fields]) OR ("Bone Transplantation"[MeSH Terms] OR ("bone"[All Fields] AND "transplantation"[All Fields]) OR "Bone Transplantation"[All Fields]) | 177,225 | 23:21:37 |
| 17 | ("Hydroxyapatites"[Mesh]) OR (Hydroxyapatites) | Most Recent |  | "Hydroxyapatites"[MeSH Terms] OR "durapatite"[MeSH Terms] OR "durapatite"[All Fields] OR "hydroxyapatite"[All Fields] OR "Hydroxyapatites"[MeSH Terms] OR "Hydroxyapatites"[All Fields] | 35,197 | 23:20:51 |
| 16 | Arthrodesis | Most Recent |  | "arthrodesed"[All Fields] OR "arthrodesing"[All Fields] OR "arthrodesis"[MeSH Terms] OR "arthrodesis"[All Fields] OR "arthrodese"[All Fields] OR "arthrodeses"[All Fields] | 45,023 | 23:17:41 |
| 15 | "Arthrodesis"[Mesh] | Most Recent |  | "Arthrodesis"[MeSH Terms] | 40,034 | 23:17:22 |
| 14 | "lumbar Vertebrae"[Mesh] | Most Recent |  | "lumbar Vertebrae"[MeSH Terms] | 59,198 | 23:12:09 |
| 13 | "Thoracic Vertebrae"[Mesh] | Most Recent |  | "Thoracic Vertebrae"[MeSH Terms] | 22,783 | 23:11:52 |
| 12 | thoracic vertebra | Most Recent |  | "thoracic vertebrae"[MeSH Terms] OR ("thoracic"[All Fields] AND "vertebrae"[All Fields]) OR "thoracic vertebrae"[All Fields] OR ("thoracic"[All Fields] AND "vertebra"[All Fields]) OR "thoracic vertebra"[All Fields] | 29,054 | 23:11:07 |
| 11 | lumbar vertebrae | Most Recent |  | "lumbar vertebrae"[MeSH Terms] OR ("lumbar"[All Fields] AND "vertebrae"[All Fields]) OR "lumbar vertebrae"[All Fields] | 64,622 | 23:10:34 |
| 10 | spine | Most Recent |  | "spine"[MeSH Terms] OR "spine"[All Fields] OR "spines"[All Fields] OR "spine s"[All Fields] | 279,288 | 23:09:49 |
| 9 | spine fracture | Most Recent |  | "spinal fractures"[MeSH Terms] OR ("spinal"[All Fields] AND "fractures"[All Fields]) OR "spinal fractures"[All Fields] OR ("spine"[All Fields] AND "fracture"[All Fields]) OR "spine fracture"[All Fields] | 37,994 | 23:09:40 |
| 8 | "Spine"[Mesh] | Most Recent |  | "Spine"[MeSH Terms] | 161,029 | 23:09:24 |
| 7 | Bone Transplantation |  |  | "bone transplantation"[MeSH Terms] OR ("bone"[All Fields] AND "transplantation"[All Fields]) OR "bone transplantation"[All Fields] | 155,373 | 23:07:59 |
| 6 | bone grafting |  |  | "bone transplantation"[MeSH Terms] OR ("bone"[All Fields] AND "transplantation"[All Fields]) OR "bone transplantation"[All Fields] OR ("bone"[All Fields] AND "grafting"[All Fields]) OR "bone grafting"[All Fields] | 163,581 | 23:07:46 |
| 5 | "Bone Transplantation"[Mesh] | Most Recent |  | "Bone Transplantation"[MeSH Terms] | 33,709 | 23:05:03 |
| 4 | bone graft | Most Recent |  | "bone transplantation"[MeSH Terms] OR ("bone"[All Fields] AND "transplantation"[All Fields]) OR "bone transplantation"[All Fields] OR ("bone"[All Fields] AND "graft"[All Fields]) OR "bone graft"[All Fields] | 171,247 | 23:03:19 |
| 3 | Hydroxyapatites | Most Recent |  | "durapatite"[MeSH Terms] OR "durapatite"[All Fields] OR "hydroxyapatite"[All Fields] OR "hydroxyapatites"[MeSH Terms] OR "hydroxyapatites"[All Fields] | 35,197 | 23:02:22 |
| 1 | "Hydroxyapatites"[Mesh] | Most Recent |  | "Hydroxyapatites"[MeSH Terms] | 22,114 | 22:59:26 |
